# Supplementary material for: The REST remodeling complex protects genomic integrity during embryonic neurogenesis
Source: eLife. 2016 Jan 8;5:e09584. doi: 10.7554/eLife.09584 (PMC4728133; doi:10.7554/eLife.09584)
Supplement: Supplementary file 1. — DOI: http://dx.doi.org/10.7554/eLife.09584.018 [file elife-09584-supp1.docx]

| **Supplementary file 1. Genes significantly upregulated in E12.5 *Cre+, Rest ^GTi/GTi^* brain vs *Rest ^GTi/GTi^* brain, revealed by microarray analyses.** | | | | | | | | | | | | |
| --- | --- | --- | --- | --- | --- | --- | --- | --- | --- | --- | --- | --- |
| **GenBank** | **Symbol** | **Description** | | | | | **Fold Change** | | | | **P-value** | |
| NM_009263 | Spp1 | secreted phosphoprotein 1 | | | 8.193 | | | | | 2.48E-005 | | |
| AV066625 | Lyz1 | lysozyme 1 | | | 4.366 | | | | | 3.27E-004 | | |
| NM_027209 | Ms4a6b | membrane-spanning 4-domains, subfamily A, member 6B | | | 4.305 | | | | | 3.39E-005 | | |
| AV246296 | Eda2r | ectodysplasin A2 isoform receptor | | | 3.874 | | | | | 2.48E-005 | | |
| AV058500 | Lyz2 | lysozyme 2 | | | 3.697 | | | | | 1.10E-003 | | |
| NM_009777 | C1qb | complement component 1, q subcomponent, beta polypeptide | | | 3.600 | | | | | 3.09E-005 | | |
| NM_011662 | Tyrobp | TYRO protein tyrosine kinase binding protein | | | 3.407 | | | | | 2.48E-005 | | |
| NM_007574 | C1qc | complement component 1, q subcomponent, C chain | | | 3.389 | | | | | 3.04E-005 | | |
| BB111335 | C1qb | complement component 1, q subcomponent, beta polypeptide | | | 3.025 | | | | | 3.36E-005 | | |
| NM_007572 | C1qa | complement component 1, q subcomponent, alpha polypeptide | | | 2.940 | | | | | 3.39E-005 | | |
| AK019319 | Apoe | apolipoprotein E | | | 2.847 | | | | | 3.40E-005 | | |
| BB333624 | C3ar1 | complement component 3a receptor 1 | | | 2.834 | | | | | 4.06E-005 | | |
| NM_010188 | Fcgr3 | Fc receptor, IgG, low affinity III | | | 2.816 | | | | | 2.38E-004 | | |
| NM_026835 | Ms4a6d | membrane-spanning 4-domains, subfamily A, member 6D | | | 2.731 | | | | | 1.64E-003 | | |
| NM_021451 | Pmaip1 | phorbol-12-myristate-13-acetate-induced protein 1 | | | 2.559 | | | | | 5.93E-003 | | |
| AW208566 | Lyz2 | lysozyme 2 | | | 2.558 | | | | | 1.88E-003 | | |
| L20315 | Mpeg1 | macrophage expressed gene 1 | | | 2.485 | | | | | 1.98E-003 | | |
| BC016551 | Fcrls | Fc receptor-like S, scavenger receptor | | | 2.465 | | | | | 7.68E-006 | | |
| NM_019467 | Aif1 | allograft inflammatory factor 1 | | | 2.463 | | | | | 1.57E-003 | | |
| NM_010745 | Ly86 | lymphocyte antigen 86 | | | 2.456 | | | | | 3.39E-005 | | |
| NM_026835 | Ms4a6d | membrane-spanning 4-domains, subfamily A, member 6D | | | 2.381 | | | | | 1.64E-003 | | |
| AI323359 | Csf1r | colony stimulating factor 1 receptor | | | 2.362 | | | | | 3.04E-005 | | |
| NM_010185 | Fcer1g | Fc receptor, IgE, high affinity I, gamma polypeptide | | | 2.357 | | | | | 3.27E-004 | | |
| NM_020008 | Clec7a | C-type lectin domain family 7, member a | | | 2.280 | | | | | 2.74E-003 | | |
| BC005569 | Rnase4 | ribonuclease, RNase A family 4 | | | 2.238 | | | | | 1.88E-003 | | |
| BC012653 | Cx3cr1 | chemokine (C-X3-C) receptor 1 | | | 2.216 | | | | | 1.97E-003 | | |
| NM_013750 | Phlda3 | pleckstrin homology-like domain, family A, member 3 | | | 2.160 | | | | | 3.56E-003 | | |
| BC001991 | Sepp1 | selenoprotein P, plasma, 1 | | | 2.159 | | | | | 1.93E-002 | | |
| NM_011210 | Ptprc | protein tyrosine phosphatase, receptor type, C | | | 2.128 | | | | | 2.81E-003 | | |
| AI385586 | Rnase4 | ribonuclease, RNase A family 4 | | | 2.115 | | | | | 4.49E-003 | | |
| BB218107 | Laptm5 | lysosomal-associated protein transmembrane 5 | | | 2.102 | | | | | 7.18E-003 | | |
| NM_007651 | Cd53 | CD53 antigen | | | 2.094 | | | | | 1.51E-002 | | |
| NM_011823 | Gpr34 | G protein-coupled receptor 34 | | | 2.081 | | | | | 4.18E-003 | | |
| NM_010708 | Lgals9 | lectin, galactose binding, soluble 9 | | | 2.080 | | | | | 4.26E-003 | | |
| AK007630 | Cdkn1a | cyclin-dependent kinase inhibitor 1A (P21) | | | 2.078 | | | | | 3.16E-004 | | |
| NM_009982 | Ctsc | cathepsin C | | | 2.069 | | | | | 1.25E-002 | | |
| BC024402 | Ms4a7 | membrane-spanning 4-domains, subfamily A, member 7 | | | 2.058 | | | | | 1.19E-002 | | |
| NM_009779 | C3ar1 | complement component 3a receptor 1 | | | 2.022 | | | | | 3.27E-004 | | |
| BG084230 | Pycard | PYD and CARD domain containing | | | 1.992 | | | | | 1.50E-002 | | |
| NM_021281 | Ctss | cathepsin S | | | 1.969 | | | | | 4.23E-003 | | |
| U66888 | Emr1 | EGF-like module containing, mucin-like, hormone receptor-like sequence 1 | | | 1.943 | | | | | 4.53E-004 | | |
| NM_007669 | Cdkn1a | cyclin-dependent kinase inhibitor 1A (P21) | | | 1.941 | | | | | 3.16E-004 | | |
| BG065754 | Ccng1 | cyclin G1 | | | 1.932 | | | | | 6.99E-003 | | |
| NM_010161 | Evi2a | ecotropic viral integration site 2a | | | 1.927 | | | | | 2.14E-002 | | |
| AV225808 | Hexb | hexosaminidase B | | | 1.911 | | | | | 1.32E-003 | | |
| NM_023044 | Slc15a3 | solute carrier family 15, member 3 | | | 1.893 | | | | | 1.76E-003 | | |
| NM_007801 | Ctsh | cathepsin H | | | 1.871 | | | | | 3.27E-004 | | |
| AK018713 | Cyba | cytochrome b-245, alpha polypeptide | | | 1.861 | | | | | 7.83E-003 | | |
| BG065754 | Ccng1 | cyclin G1 | | | 1.807 | | | | | 8.24E-004 | | |
| **GenBank** | **Symbol** | **Description** | | **Fold Change** | | | | | | | | **P- value** |
| NM_010422 | Hexb | hexosaminidase B | 1.797 | | | | | | 1.45E-003 | | | |
| BG065754 | Ccng1 | cyclin G1 | | | | 1.776 | | 5.43E-003 | | | | |
| AK021181 | Myo1f | myosin IF | | | | 1.766 | | 3.42E-002 | | | | |
| AI323359 | Csf1r | colony stimulating factor 1 receptor | | | | 1.763 | | 3.27E-004 | | | | |
| NM_013602 | Mt1 | metallothionein 1 | | | | 1.757 | | 4.86E-002 | | | | |
| AI451985 | Mapkapk3 | mitogen-activated protein kinase-activated protein kinase 3 | | | | 1.755 | | 4.56E-002 | | | | |
| NM_011175 | Lgmn | legumain | | | | 1.746 | | 3.27E-004 | | | | |
| NM_019388 | Cd86 | CD86 antigen | | | | 1.743 | | 3.45E-002 | | | | |
| AI385586 | Rnase4 | ribonuclease, RNase A family 4 | | | | 1.713 | | 4.67E-003 | | | | |
| M86736 | Grn | granulin | | | | 1.705 | | 1.25E-002 | | | | |
| BB157866 | Fyb | FYN binding protein | | | | 1.702 | | 1.27E-002 | | | | |
| AI326478 | Igh-6 | immunoglobulin heavy chain 6 (heavy chain of IgM) | | | | 1.700 | | 7.29E-003 | | | | |
| M14222 | Ctsb | cathepsin B | | | | 1.684 | | 1.30E-003 | | | | |
| BC025220 | Pqlc3 | PQ loop repeat containing | | | | 1.677 | | 3.30E-002 | | | | |
| NM_009779 | C3ar1 | complement component 3a receptor 1 | | | | 1.664 | | 2.39E-002 | | | | |
| BC027331 | P2ry6 | pyrimidinergic receptor P2Y, G-protein coupled, 6 | | | | 1.654 | | 1.14E-002 | | | | |
| NM_021351 | Cryba4 | crystallin, beta A4 | | | | 1.652 | | 3.02E-002 | | | | |
| AV166504 | Grn | granulin | | | | 1.639 | | 8.87E-003 | | | | |
| AF181829 | Plek | pleckstrin | | | | 1.631 | | 4.18E-003 | | | | |
| U29539 | Laptm5 | lysosomal-associated protein transmembrane 5 | | | | 1.627 | | 1.56E-002 | | | | |
| NM_009983 | Ctsd | cathepsin D | | | | 1.626 | | 1.30E-003 | | | | |
| BB542535 | Ctsh | cathepsin H | | | | 1.625 | | 1.30E-003 | | | | |
| BB000455 | Grn | granulin | | | | 1.622 | | 9.34E-003 | | | | |
| NM_011337 | Ccl3 | chemokine (C-C motif) ligand 3 | | | | 1.615 | | 1.64E-003 | | | | |
| U05264 | Lilrb4 | leukocyte immunoglobulin-like receptor, subfamily B, member 4 | | | | 1.593 | | 1.93E-002 | | | | |
| AK005731 | 1700007K13Rik | RIKEN cDNA 1700007K13 gene | | | | 1.589 | | 2.57E-002 | | | | |
| NM_026405 | Rab32 | RAB32, member RAS oncogene family | | | | 1.588 | | 4.83E-002 | | | | |
| M14222 | Ctsb | cathepsin B | | | | 1.578 | | 5.71E-004 | | | | |
| NM_008419 | Kcna5 | potassium voltage-gated channel, shaker-related subfamily, member 5 | | | | 1.569 | | 2.19E-002 | | | | |
| NM_010877 | Ncf2 | neutrophil cytosolic factor 2 | | | | 1.565 | | 1.45E-003 | | | | |
| BB226392 | Igh-6 | immunoglobulin heavy chain 6 (heavy chain of IgM) | | | | 1.556 | | 1.14E-002 | | | | |
| AF181829 | Plek | pleckstrin | | | | 1.550 | | 3.92E-002 | | | | |
| M14222 | Ctsb | cathepsin B | | | | 1.546 | | 7.45E-004 | | | | |
| BC025083 | Glipr1 | GLI pathogenesis-related 1 (glioma) | | | | 1.541 | | 6.62E-003 | | | | |
| AV308638 | Sesn2 | sestrin 2 | | | | 1.541 | | 2.34E-002 | | | | |
| AF143181 | Fcgr1 | Fc receptor, IgG, high affinity I | | | | 1.525 | | 6.81E-003 | | | | |
| BM238906 | Nckap1l | NCK associated protein 1 like | | | | 1.506 | | 1.50E-002 | | | | |
| NM_010368 | Gusb | glucuronidase, beta | | | | 1.504 | | 4.35E-002 | | | | |
| AI844633 | Ncf1 | neutrophil cytosolic factor 1 | | | | 1.492 | | 3.89E-002 | | | | |
| NM_010077 | Drd2 | dopamine receptor 2 | | | | 1.481 | | 2.30E-003 | | | | |
| AF220015 | Trim30 | tripartite motif-containing 30 | | | | 1.442 | | 4.83E-002 | | | | |
| NM_019521 | Gas6 | growth arrest specific 6 | | | | 1.437 | | 2.91E-002 | | | | |
| NM_054087 | Slc19a2 | solute carrier family 19 (thiamine transporter), member 2 | | | | 1.435 | | 1.76E-002 | | | | |
| BC012247 | Dcxr | dicarbonyl L-xylulose reductase | | | | 1.415 | | 2.66E-002 | | | | |
| AF440692 | Trf | transferrin | | | | 1.398 | | 4.35E-002 | | | | |
| BB449198 | Slc19a2 | solute carrier family 19 (thiamine transporter), member 2 | | | | 1.393 | | 2.21E-002 | | | | |
| NM_022325 | Ctsz | cathepsin Z | | | | 1.373 | | 6.14E-003 | | | | |
| NM_022325 | Ctsz | cathepsin Z | | | | 1.373 | | 1.56E-002 | | | | |
| AV375176 | Centd1 | centaurin, delta 1 | | | | 1.372 | | 2.27E-002 | | | | |
| BB435348 | Lrp11 | low density lipoprotein receptor-related protein 11 | | | | 1.364 | | 2.44E-002 | | | | |
| NM_023422 | Hist1h2bc | histone cluster 1, H2bc | | | | 1.363 | | 1.27E-002 | | | | |
| **GenBank** | **Symbol** | **Description** | | | | **Fold Change** | | **P- value** | | | | |
| NM_022325 | Ctsz | cathepsin Z | | | | 1.346 | | 6.14E-003 | | | | |
| BB741897 | Rsad2 | radical S-adenosyl methionine domain containing 2 | | | | 1.321 | | 2.38E-002 | | | | |
| NM_009121 | Sat1 | spermidine/spermine N1-acetyl transferase 1 | | | | 1.314 | | 4.76E-002 | | | | |
| U07631 | Hexa | hexosaminidase A | | | | 1.299 | | 3.57E-002 | | | | |
|  |  |  | | | |  | |  | | | | |

microglia sensome

p53-dependent apoptosis

other

neuronal

References

1. Hickman, S.E.*, et al.* The microglial sensome revealed by direct RNA sequencing. *Nat Neurosci* **16**, 1896-1905.

2. Butovsky, O.*, et al.* Identification of a unique TGF-beta-dependent molecular and functional signature in microglia. *Nat Neurosci* **17**, 131-143.

3. Liang, Y.*, et al.* Expression profiling of Rab GTPases reveals the involvement of Rab20 and Rab32 in acute brain inflammation in mice. *Neurosci Lett* **527**, 110-114.

4. Origasa, M.*, et al.* Activation of a novel microglial gene encoding a lysosomal membrane protein in response to neuronal apoptosis. *Brain Res Mol Brain Res* **88**, 1-13 (2001).
