# Supplementary material for: The REST remodeling complex protects genomic integrity during embryonic neurogenesis
Source: eLife. 2016 Jan 8;5:e09584. doi: 10.7554/eLife.09584 (PMC4728133; doi:10.7554/eLife.09584)
Supplement: Supplementary file 2. — DOI: http://dx.doi.org/10.7554/eLife.09584.019 [file elife-09584-supp2.docx]

| **Supplementary File 2. Genes significantly upregulated in brains of E12.5 *Cre+, Rest ^GTi/GTi^ p53 ^fl/fl^* mice vs E12.5 Rest *^GTi/GTi^ Trp53 ^fl/fl^* revealed by microarray analyses.** | | | | |
| --- | --- | --- | --- | --- |
| **GenBank** | **Symbol** | **Description** | **Fold change** | **P-value** |
| NM_007532 | Bcat1 | Branched chain aminotransferase 1 | 1.6 | 3.06E-02 |
| NM_010471 | Hpca | C57BL/6J hippocalcin | 1.46 | 9.68E-03 |
| NM_009130 | Scg3 | Secretogranin III | 1.41 | 9.39E-03 |
| NM_007693 | Chga | Chromogranin A | 1.4 | 1.49E-02 |
| NM_013710 | Fgd2 | FYVE, RhoGEF and PH domain containing 2 | 1.4 | 4.70E-02 |
| NM_024185 | 2310047O13Rik | RIKEN cDNA 2310047O13 gene, mRNA | 1.38 | 3.48E-03 |
| NM_027139 | Taf9 | TAF9 RNA polymerase II, TATA box binding protein (TBP)-associated factor | 1.38 | 4.31E-02 |
| NM_007756 | Cplx1 | Complexin 1 | 1.38 | 5.27E-03 |
| NM_021295 | Lancl1 | LanC (bacterial lantibiotic synthetase component C)-like 1 | 1.38 | 4.16E-03 |
| NM_021433 | Stx6 | Syntaxin 6 | 1.37 | 3.53E-02 |
| NM_026246 | Mrpl49 | Mitochondrial ribosomal protein L49) | 1.36 | 1.01E-04 |
| NM_011992 | Rcn2 | Reticulocalbin 2 | 1.36 | 9.29E-03 |
| NM_025512 | Zfand1 | Zinc finger, AN1-type domain 1 | 1.35 | 3.68E-02 |
| NM_172379 | Fam149b | Family with sequence similarity 149, member B | 1.35 | 2.34E-02 |
| XM_485277 | Scnm1 | Sodium channel modifier | 1.35 | 1.64E-02 |
| NM_011484 | Stam | Signal transducing adaptor molecule (SH3 domain and ITAM motif) 1 | 1.35 | 3.24E-02 |
| NM_025507 | Snw1 | SNW domain containing 1 (Snw1) | 1.33 | 1.31E-02 |
| NM_010435 | Hira | Histone cell cycle regulation defective homolog | 1.33 | 4.78E-02 |
| NM_010267 | Gdap1 | Ganglioside-induced differentiation-associated-protein 1 | 1.32 | 2.52E-02 |
| NM_021408 | Ush2a | Usher syndrome 2A (autosomal recessive, mild) homolog | 1.31 | 4.10E-02 |
| NM_020018 | Magea5 | Melanoma antigen, family A, 5 | 1.31 | 3.53E-02 |
| XM_126221 | Neural4 | neuralized homolog 4 (Drosophila) | 1.31 | 3.37E-02 |
| NM_172401 | 1110057K04Rik | RIKEN cDNA 1110057K04 gene | 1.31 | 3.39E-02 |
| NM_020585 | Golga7 | Golgi autoantigen, golgin subfamily a, 7 | 1.31 | 3.12E-02 |
| NM_025701 | Trappc5 | Trafficking protein particle complex 5 (Trappc5) | 1.31 | 3.41E-02 |
| NM_133999 | Fig4 | FIG4 homolog (S. cerevisiae) | 1.31 | 4.11E-02 |
| NM_008950 | Psmc5 | Protease (prosome, macropain) 26S subunit, ATPase 5 | 1.31 | 1.78E-02 |
| NM_172851 | Cntnap5b | Contactin associated protein-like 5B | 1.3 | 3.73E-03 |
| NM_001037741 | Gpx4 | Phospholipid hydroperoxide glutathione peroxidase | 1.29 | 4.16E-02 |
| NM_009996 | Cyp24a1 | Cytochrome P450, family 24, subfamily a, polypeptide 1 | 1.29 | 1.65E-02 |
| NM_008510 | Xcl1 | Chemokine (C motif) ligand 1 | 1.29 | 3.32E-02 |
| NM_021511 | Rrs1 | RRS1 ribosome biogenesis regulator homolog | 1.29 | 3.42E-02 |
| XM_131022 | Them4 | thioesterase superfamily member 4 | 1.29 | 4.87E-03 |
| NM_133797 | 4833439L19Rik | RIKEN cDNA 4833439L19 gene | 1.29 | 1.21E-02 |
| NM_194350 | Mafa | V-maf musculoaponeurotic fibrosarcoma oncogene family | 1.29 | 1.75E-02 |
| NM_146983 | Olfr1256 | Olfactory receptor 1256 | 1.29 | 4.29E-02 |
| NM_172697 | Prpf38a | PRP38 pre-mRNA processing factor 38 (yeast) | 1.29 | 3.65E-02 |
| NM_001013759 | Gas2l2 | Growth arrest-specific 2 like 2 | 1.28 | 5.12E-03 |
| NM_001007573 | Maneal | Gene model 50 | 1.28 | 3.82E-03 |
| **GenBank** | **Symbol** | **Description** | **Fold change** | **P-value** |
| NM_145404 | Prmt7 | Protein arginine N-methyltransferase 7 (Prmt7) | 1.28 | 2.82E-02 |
| NM_022325 | Ctsz | Cathepsin Z | 1.28 | 3.78E-03 |
| NM_001081233 | EG433923 | Predicted gene, EG433923 | 1.28 | 1.83E-02 |
| XM_111253 | Fbll1 | fibrillarin-like 1 | 1.28 | 1.80E-02 |
| NM_146975 | Olfr1273 | Olfactory receptor 1273 (Olfr1273) | 1.28 | 1.92E-02 |
| NM_207140 | Olfr1212 | Olfactory receptor 1212 (Olfr1212) | 1.28 | 1.65E-02 |
| NM_144858 | Dus3l | Dihydrouridine synthase 3-like (S. cerevisiae) | 1.27 | 9.77E-03 |
| NM_177153 | - | C230009H10Rik | 1.27 | 4.32E-02 |
| NM_010360 | Gstm5 | Glutathione S-transferase, mu 5 | 1.27 | 9.50E-03 |
| NM_198414 | Paqr9 | Progestin and adipoQ receptor family member IX | 1.27 | 4.26E-02 |
| NM_181582 | Eif5a | Eukaryotic translation initiation factor 5A | 1.27 | 1.99E-03 |
| NM_010813 | Mnt | Max binding protein | 1.27 | 3.47E-02 |
| NM_172735 | Zc3hc1 | Zinc finger, C3HC type 1 | 1.27 | 4.84E-03 |
| NM_144532 | Cabp4 | Calcium binding protein 4 | 1.27 | 1.05E-02 |
| NM_010647 | Klra7 | Killer cell lectin-like receptor subfamily A, member 12 | 1.27 | 8.40E-03 |
| NM_053193 | Cpsf1 | Cleavage and polyadenylation specific factor 1 | 1.27 | 5.80E-03 |
| NM_134021 | Pnpo | Pyridoxine 5-phosphate oxidase | 1.26 | 2.41E-02 |
| NM_013732 | Cartpt | CART prepropeptide transcript variant 1 | 1.26 | 2.59E-02 |
| NM_025359 | Tspan13 | Tetraspanin 13 | 1.26 | 3.29E-02 |
| NM_080437 | Celsr3 | Cadherin, EGF LAG seven-pass G-type receptor 3 | 1.26 | 3.02E-02 |
| NM_008063 | Slc37a4 | Solute carrier family 37 (glucose-6-phosphate transporter), member 4 | 1.26 | 4.08E-02 |
| NM_009133 | Stmn3 | Stathmin-like 3 | 1.26 | 2.94E-02 |
| NM_019926 | Mtm1 | X-linked myotubular myopathy gene 1 | 1.26 | 4.14E-03 |
| NM_025279 | Hnrnpk | Heterogeneous nuclear ribonucleoprotein K | 1.26 | 1.04E-02 |
| NM_145511 | BC003331 | CDNA sequence BC003331 | 1.26 | 4.05E-02 |
| NM_008338 | Ifngr2 | Interferon gamma receptor 2 | 1.26 | 3.75E-02 |
| NM_026408 | Sncaip | Synuclein, alpha interacting protein | 1.26 | 1.23E-02 |
| NM_026529 | 2700062C07Rik | RIKEN cDNA 2700062C07 gene | 1.25 | 2.88E-02 |
| NM_153385 | Clrn1 | Clarin 1 transcript variant 2 | 1.25 | 3.55E-02 |
| NM_030007 | - | A430107J06Rik | 1.25 | 4.71E-02 |
| NM_021510 | Hnrnph1 | Heterogeneous nuclear ribonucleoprotein H1 | 1.25 | 3.10E-02 |
| NM_172916 | Hydin | Hydrocephalus inducing, | 1.25 | 4.49E-02 |
| NM_008073 | Gabrg2 | Gamma-aminobutyric acid receptor, subunit gamma 2 | 1.25 | 4.76E-02 |
| NM_025292 | Synj2bp | Activin receptor interacting protein 2 | 1.25 | 8.02E-03 |
| XM_181304 | Sat1 | spermidine/spermine N1-acetyl transferase 2 | 1.25 | 4.14E-02 |
| NM_023732 | Abcb6 | ATP-binding cassette, sub-family B (MDR/TAP), member 6 (Abcb6), nuclear gene encoding mitochondrial protein | 1.25 | 1.00E-03 |
| NM_178589 | Tnfrsf21 | Tumor necrosis factor receptor superfamily, member 21 | 1.25 | 2.55E-02 |
| NM_029831 | 1700127D06Rik | RIKEN cDNA | 1.25 | 2.10E-02 |
| NM_028776 | Scyl3 | SCY1-like 3 (S. cerevisiae | 1.25 | 3.26E-02 |
| NM_007566 | Birc6 | Baculoviral IAP Repeat-Containing 6 | 1.25 | 4.39E-03 |
| NM_147050 | Olfr659 | Olfactory receptor 659 | 1.25 | 2.83E-02 |
| NM_009860 | Cdc25c | Cell division cycle 25 homolog C (S. pombe) | 1.25 | 7.67E-03 |
| **GenBank** | **Symbol** | **Description** | **Fold change** | **P-value** |
|  |  |  |  |  |
| NM_138757 | 4933424B01Rik | RIKEN cDNA 4933424B01 gene (4933424B01Rik) | 1.24 | 1.00E-02 |
| NM_178642 | Ano1 | Anoctamin 1, calcium activated chloride channel | 1.24 | 3.33E-02 |
| NM_173398 | Gpr171 | G protein-coupled receptor 171 | 1.24 | 3.04E-02 |
| NM_008092 | Gata4 | Transcription factor GATA-4 | 1.24 | 2.98E-02 |
| NM_001080943 | Zdhhc22 | Zinc finger, DHHC-type containing 22 | 1.24 | 4.64E-03 |
| NM_027013 | Scnm1 | Sodium channel modifier 1 | 1.24 | 4.40E-02 |
| NM_145415 | AA408296 | Expressed sequence | 1.24 | 4.02E-02 |
| NM_175454 | C630004H02Rik | RIKEN cDNA C630004H02 gene | 1.24 | 3.05E-02 |
| NM_026162 | Plxdc2 | Plexin domain containing 2 | 1.24 | 7.80E-03 |
| NM_008131 | Glul | Glutamine synthetase (Glul) | 1.23 | 4.96E-02 |
| NM_013668 | Kdm5c | Jumonji, AT rich interactive domain 1C (Rbp2 like) | 1.23 | 2.14E-02 |
|  |  |  |  |  |
|  |  |  |  |  |
|  | dfdfszcmvkdgs | Genes with REST binding sites within 10kb from the TSS^1^ |  |  |

References

1. McGann, J.C.*, et al.* Polycomb- and REST-associated histone deacetylases are independent pathways toward a mature neuronal phenotype. *Elife* **3**, e04235.
