# Supplementary material for: The REST remodeling complex protects genomic integrity during embryonic neurogenesis
Source: eLife. 2016 Jan 8;5:e09584. doi: 10.7554/eLife.09584 (PMC4728133; doi:10.7554/eLife.09584)
Supplement: Supplementary file 3. — DOI: http://dx.doi.org/10.7554/eLife.09584.020 [file elife-09584-supp3.docx]

**Supplementary File 3.**

**Primer sequences used in the study**

| **Primer name** | **5’-3’** |
| --- | --- |
| **qRT-PCR** | |
| qRestA3 (exon 1-2) | gcgcacagttcagaggagt |
| qRestB3 (exon 1-2) | tgttggcactgttgttgaaga |
| qRestA4 (exon2-3) | caagtgcaactacttctcagacaga |
| qRestB4 (exon2-3) | aaggacaaagttcacatttatacgg |
| qRestA5 (exon3-4) | gacacatgcggactcattca |
| qRestB5 (exon3-4) | gtcttgcatgtcgggtca |
| qRestA1 (exon4) | ggctgctctcaaggagtctg |
| qRestB1 (exon4) | ttctgctcagtgtccacgtc |
| q18SA | cggacacggacaggattgacaga |
| q18SB | accacccacggaatcgagaaaga |
| qp53A1 | atgcccatgctacagaggag |
| qp53B1 | agactggcccttcttggtct |
| qp21A1 | cagatccacagcgatatcca |
| qp21B1 | ggcacactttgctcctgtg |
| qCyclinG1A1 | tggacagattcttgtctaaaatgaag |
| qCyclinG1B1 | cagtgggacattcctttcctc |
| qSestrin2A1 | acatccactgcgtctttgg |
| qSestrin2B1 | cgtcttgatatagattttgaggttcc |
| qPmaipA1 | cagatgcctgggaagtcg |
| qPmaipB1 | tgagcacactcgtccttcaa |
| qBtg2A1 | gcgagcagagactcaaggtt |
| qBtg2B1 | ccagtggtgtttgtaatgatcg |
| qPerpA1 | gaccccagatgcttgttttc |
| qPerpB1 | accagggagatgatctggaa |
| qGlraA1 | tgcctgctcttcgtgttctct |
| qGlraB1 | tgttgccgagacacaaagtt |
| qSnap25A1 | acgggagcagatggcca |
| qSnap25B1 | cccgggcatcgtttgtt |
| qSynaptophysinA1 | CCTGTCCGATGTGAAGATGG |
| qSynaptophysinB1 | TTCAGGAAGCCAAACACCAC |
| qTuJA1 | gcgcctttggacacctattca |
| qTuJB1 | ccgcgccctccgtatagtgc |
| qSox2A | tccaaaaactaatcacaacaatcg |
| qSox2B | gaagtgcaattgggatgaaaa |
| qCdkn2cA1 | aaatggaattgggagaactgc |
| qCdkn2cB1 | aaattgggattagcacctctg |
| qVgfA | ACTCCAGCCACGGAACAGAGC |
| qVgfB | ATGACCAACGGGCTGCCAGAGAC |
| qAp3b2A | cacggtgagatcatcaaacact |
| qAp3b2B | cagaggatgctggctcgt |
| qCD68A | gacctacatcagagcccgagt |
| qCD68B | cgccatgaatgtccactg |
| qCD44A | tccttctttatccggagcac |
| qCd44B | cctggagtccttggatgagt |
| qMetA | gctctggaggacaagaccac |
| qMetB | ttctgctacaccgtcagcttt |
| qNf1A | ttctaccaagctggcacctc |
| qNf1B | tctggccagttttgaacctc |
| qTraddA | tgaattacatcttagcccagaagc |
| qTraddB | cacacgtcagtttgcagagc |
| qChi3l1A | gaccctggcctactacgaga |
| qChi3l1B | ttggtagcgaagggaacct |
| qMertkA | gatggttctggccccact |
| qMertkB | ctgatctagctcggtctcttcc |
| qRelbA | gtgacctctcttccctgtcact |
| qRelbB | tgtattcgtcgatgatttccaa |
| qJag1A | GAGGCGTCCTCTGAAAAACA |
| qJag1B | ACCCAAGCCACTGTTAAGACA |
| qNotch3A | agctgggtcctgaggtgat |
| qNotch3B | agacagagccggttgtcaat |
| qGli2A | gcagactgcaccaaggagtatgac |
| qGli2B | cgtggatgtgttcattgttgatatga |
| qFgrg3A | cttttggctgcgtgttca |
| qFgfr3B | tgccagcctcatcagtttc |
| qEgfrA | caacaaagaaatccttgacgaa |
| qEgfrB | ggacagtggaggtcagacaga |
| qAkt2A | tcgtgtggcaggatgtgtat |
| qAkt2B | acctggtgtcagtctcagagg |
| qCD133A | gaaaagttgctctgcgaacc |
| qCD133B | ctcgacctcttttgcaatcc |
| qNestinA | CTGCAGGCCACTGAAAAGTT |
| qNestinB | TCTGACTCTGTAGACCCTGCTTC |
| qDll3A | acatcgaagcccgtagaatc |
| qDll3B | gggggcagctgtagtgaa |
| qNkx2.2A | agggcttaagatgcctggat |
| qNkx2.2B | tgtcccagagtcacggagt |
| qDcxA | agctgactcaggtaacgacca |
| qDcxB | GCTTTGACTTAGGTGTTGAGAGC |
| qOlig2A | AGACCGAGCCAACACCAG |
| qOlig2B | AAGCTCTCGAATGATCCTTCTTT |
| qErbb3A | ctgccacgagaactgcac |
| qErbb3B | tgcttggcctaaacagtcttg |
| qPdgfraA | gccctgtgaggagacagc |
| qPdgfraB | gcaaattgacatagaaggagaag |
| qMash1A | tctcctgggaatggactttg |
| qMash1B | cgttggcgagaaacactaaag |
| qFbx03A | gggtgtctatagctcgattgga |
| qFbx03B | cggattcatccatgtctgc |
| qSncgA | caacacagtggccaacaaga |
| qSncgB | ggggttccaagtcctcctt |
| qNeflA | ccctctgaaggagaagcaga |
| qNeflB | tcttttgtgtcttcagactcatcc |
| qGabra1A | cttctgctacaaccactgaacg |
| qGabra1B | gcccactaaaattcggaagc |
| qNg2A | CCCAAGCTCTAGGACGTACCT |
| qNg2B | GGCGGTAAGCTACATCTGGTTC |
| qMyt1A | gcccactctgctgacctc |
| qMyt1B | tgaagcatagttccctgttatgtg |
| qSlc12a5A | ccacttacgcattactgcagag |
| qSlc12a5B | cgtaggtgtatgccgagatg |
| qGuk1A | gacagacatggagagcagca |
| qGuk1B | tgcttcagggttgcatagg |
| qSyt1A | agggggtcttccctttttaag |
| qSyt1B | cggatgttggttgttcgag |
| qTtpA | gctcgccatttataatagtttttca |
| qTtpB | tcacagacgctttcctgct |
| qSlc12a5A | ccacttacgcattactgcagag |
| qSlc12a5B | cgtaggtgtatgccgagatg |
| **qChIP** | TGACACCTCGTCCCTCTACTCC |
| ChIPGlraA1 | gcacttacgaagccagagagc |
| ChIPGlraB1 | TGACACCTCGTCCCTCTACTCC |
| ChIPMyf5A1 | ggttggggccctctttatatattcct |
| ChIPMyf5B1 | ctagaagaaagacaagaggcttgc |
| ChIPSnap25A | GGACGGCGATCCGGCTGCAGC |
| ChIPSnap25B | GATCTCAGAGAGAAAATTTGGCCTC |
| ChIPSnap25 CDSA | ttaagatgtcttgggtctcgtt |
| ChIPSnap25 CDSB | cagtagctctgtggaatgtcac |
| qRest genomic A | AGCCCAGGTTGATCTTGAACTC |
| qRest genomic B | TCCCAACACTCAGAATGCTGTT |
| qRest genomic βgeo A | ccggctacctgcccattc |
| qRest genomic βgeo B | aagaccggcttccatccg |
| qGapdh genomic A | ACACGCTTGGTGCGTGCAC |
| qGapdh genomic B | CGTAAAGCCGCGAGTAGCTGG |
|  |  |
|  |  |
|  |  |
|  |  |
|  |  |
|  |  |
|  |  |
|  |  |
|  |  |
|  |  |
|  |  |
|  |  |
|  |  |
|  |  |
|  |  |
|  |  |
|  |  |
|  |  |
|  |  |
|  |  |
|  |  |
|  |  |
|  |  |
|  |  |
|  |  |
|  |  |
|  |  |
|  |  |
|  |  |
|  |  |
